# Creation and annihilation of mobile fractional solitons in atomic chains

**DOI:** 10.1038/s41565-021-01042-8

**Published:** 2021-12-22

**Authors:** Jae Whan Park, Euihwan Do, Jin Sung Shin, Sun Kyu Song, Oleksandr Stetsovych, Pavel Jelinek, Han Woong Yeom

**Affiliations:** 1 Center for Artificial Low Dimensional Electronic Systems, Institute for Basic Science (IBS), Pohang, Korea; 2 Department of Physics, Pohang University of Science and Technology, Pohang, Korea; 3 Institute of Physics of the Czech Academy of Sciences, Prague, Czech Republic

**Keywords:** Topological defects, Surfaces, interfaces and thin films

## Abstract

Localized modes in one-dimensional (1D) topological systems, such as Majonara modes in topological superconductors, are promising candidates for robust information processing. While theory predicts mobile integer and fractional topological solitons in 1D topological insulators, experiments so far have unveiled immobile, integer solitons only. Here we observe fractionalized phase defects moving along trimer silicon atomic chains formed along step edges of a vicinal silicon surface. By means of tunnelling microscopy, we identify local defects with phase shifts of 2π/3 and 4π/3 with their electronic states within the band gap and with their motions activated above 100 K. Theoretical calculations reveal the topological soliton origin of the phase defects with fractional charges of ±2e/3 and ±4e/3. Additionally, we create and annihilate individual solitons at desired locations by current pulses from the probe tip. Mobile and manipulable topological solitons may serve as robust, topologically protected information carriers in future information technology.

## Abstract (web summary)

The movement of fractionalized phase defects, that can be considered as fractional solitons promising for future information technology, is observed in atomic chains formed along step edges of silicon surfaces, solitons may serve as robust, topologically protected information carriers in future information technology

## Main

Localized topological modes, such as Majorana edge modes in topological superconductors^1,2^ and skyrmion excitations in magnetic materials^3,4^, are attracting great interest as promising platforms for robust information processing^5,6^. For one-dimensional (1D) topological insulators, another kind of topological local mode, the soliton^7–10^, has been known about for a long time. Topological solitons, which have both edge-mode and excitation characteristics, have been identified not only in spontaneous 1D insulators such as polyacetylene chains^9^ and surface atomic chains^11^ but also in ultracold atoms^12–14^, photonic crystals^15–17^ and acoustic lattices^18,19^. In contrast to a Majonara edge mode, solitons can move fast with topologically protected information as in the case of an unpinned skyrmion. The technology of using solitons as the robust media of delivering information was well established in classical wave systems based on optical solitons^20,21^. In quantum mechanical systems, solitons can provide even more exciting opportunities such as the multilevel information processing^22,23^, quantum entanglements^24,25^ and the use of fractional quanta^26–28^. Among these exciting possibilities, only the multilevel information processing has been demonstrated recently by the *Z*_4_ solitons in indium atomic chains^29^. However, the soliton motion is largely prohibited by pinning defects or the interchain interaction in most 1D electronic systems^29,30^, making the realization of a mobile soliton with fractionalized quanta a long-standing challenge. Beyond observing the existence and the interaction of solitons, the generation and manipulation of individual solitons in electronic systems has to be demonstrated for many possible applications.

Among various proposals in these challenges^26–28^, trimer chains have been the most widely discussed with a particular focus on fractional charges. In 1D electronic systems of trimers, solitons are endowed with fractional charges of ±2e/3 and ±4e/3 in contrast to integer charges of solitons in conventional dimer chains due to the spin degree of freedom^7,8^. That is, trimer solitons are the simplest form of fractionalized solitons in an electronic system. Contrary to the simplicity, theoretical works reveal various exotic properties of solitons in trimer systems^31–35^. Moreover, considering the well-established ternary computing architecture^36^ and the current interest in the ternary system for low power and/or neuromorphic computing systems^37^, the use of topologically protected trimer solitons is expected to expedite exciting development in information technology. However, no electronic system with trimer solitons has been identified yet.

In this respect, the silicon atomic chains on a vicinally cut silicon crystal (Si(553)) has attracted our attention. By adsorption of a proper amount of gold atoms, a regular array of step-edge silicon chains is stabilized with unsaturated dangling bonds. This system was found to transit into a trimer structure below about 200 K (refs.^38–40^) and the existence of the phase defects was noticed with their mobility and topological nature unknown^39,41,42^. In the present work, we directly identify individual mobile solitons along these trimer atomic chains by scanning tunnelling microscopy and spectroscopy (STM and STS). We observe two different types of soliton with fractionalized (2π/3 and 4π/3) phase shifts, respectively, which are immobile at low temperature but their motion occurs above 100 K. Their solitonic property is confirmed by their in-gap electronic states and their immunity for scattering. Density functional theory (DFT) and tight-binding calculations reveal more about the topological properties of these solitons and their fractionalized charges. We also succeed to generate and annihilate a soliton on a desired location by the tunnelling electron pulse from the probe tip, making the first step towards the manipulation of individual solitons. An important step towards using mobile and robust carriers of fractional quanta is thus made.

### Mobile phase defects

**Fig. 1:**
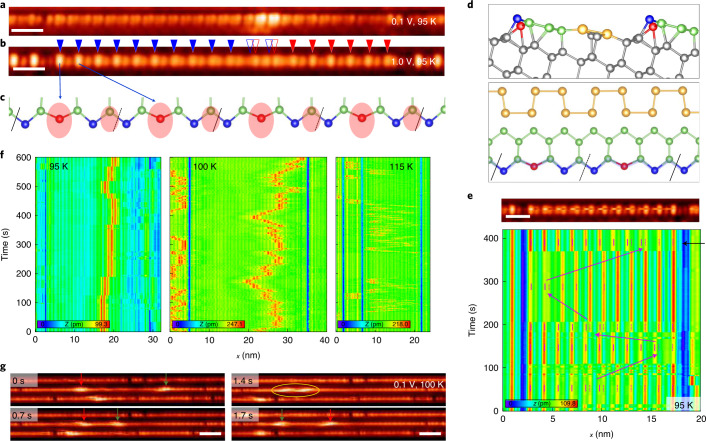
Mobile phase defects on Si chains of a Si(553)–Au surface. **a**,**b** STM images of a phase-shift defect along a Si step-edge chain in its CDW state at 95 K (sample bias voltage *V*_s_ of 0.1 (**a**) and 1.0 V (**b**); scale bars, 2 nm). Blue and red triangles denote the ×3-period CDW on the left and right domains, respectively. Empty triangles indicate the phase mismatch. **c**, Schematic atomic structure of a Si step-edge chain in the CDW state (see the blue shading in **d**). Red and blue balls represent the distorted and undistorted Si atoms at the step edge, respectively. The ovals denote the protrusions observed in STM with a bias of 1.0 V **b**. **d**, Atomic structure (side and top view) model of the periodically distorted CDW phase^40^. Yellow, green and grey balls represent Au, top-layer Si and bulk Si atoms, respectively. **e**, A continuous real time measurement of the STM profiles (*V*_s_ = 1.0 V) for the same chain segment for a time interval of about 400 s. Full and broken purple lines indicate the 3a_0_ CDW and purple arrows highlight the motion of the phase-shift defect. Top panel is a STM image (*V*_s_ = 1.0 V) of a Si chain showing a lateral shift of the CDW in the middle of the image (taken as line scans from the top to the bottom), which is related to a hopping event of a phase defect (scale bar, 2 nm). **f**, Similar real time STM profile measurements at three different temperatures but with a low bias of 0.1 eV, where the phase defect is images with a strong contrast (red in the profiles). **g**, Snapshot STM images (*V*_s_ = 0.1 V) of the mobile phase defects at 100 K (scale bars, 4 nm) (Supplementary Video 1). The arrows indicate the two particular phase defects and the ellipse indicates the temporary pairing of them.

**Fig. 2:**
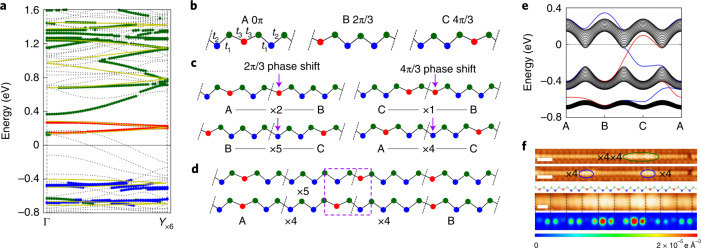
Electronic structure of the Si(553)–Au surface in its CDW state. **a**, Band dispersion along the step edge as calculated by DFT. Red, blue and green circles represent the bands localized mainly on outer distorted, outer undistorted and inner Si atoms of zigzag chain (red, blue and green atoms in Fig. 1c), respectively. Yellow lines denote the tight-binding band of the zigzag Si chain. **b**, Schematics of three translationally degenerate phases (A, B and C) for the distorted Si chain that corresponds to the step-edge zigzag Si chain marked by the blue shading in Fig. 1d. **c**, Four distinct phase boundary (defect) structures. **d**, Comparison between a ×5 and a × 4 × 4 structure with the same overall phase shift. **e**, Energy spectrum for the adiabatic evolution of open boundary trimer chain (15-unit cell). The tight-binding parameters were taken for the phase evolution of A → B → C → A. The black lines denote the eigenstates of the open boundary chain and red and blue lines correspond to localized states at right and left edges, respectively. **f**, Two successive STM images of a Si chain with two ×4 defects, paired or separated (*V*_s_ = 1.0 V at 95 K, scale bars, 2 nm) and enlarged experimental and simulated STM images of a × 4 × 4 defect (*V*_s_ = 0.1 V, scale bar, 0.5 nm).

The surface of a vicinal Si(553) crystal with an optimized coverage of Au adatoms form a well-ordered array of Si and Au atomic chains with very narrow (1.3 nm in width) terraces (Fig. 1g)^40,43,44^. Each terrace consists of double Au chains and a Si honeycomb chain on its topmost layer (Fig. 1d)^40,43,44^ (more detailed atomic structure in Supplementary Fig. 6). What concerns the present work are step-edge Si atoms with dangling bonds, which correspond to one side of the Si honeycomb chain (blue and red balls in Fig. 1c) and to the rows of bright protrusions in the STM topographs (Fig. 1b). Its low-temperature atomic structure has presented intrigue with contradictory suggestions of a charge density wave (CDW) insulator with a periodic lattice distortion^38,39^ and an antiferromagnetic insulator with a spin ordering^43^. Very recent DFT calculations found a distorted CDW structure explaining most of the experimental data^40^. Below the transition temperature of 200 K, the STM images exhibit a structural distortion in a high empty-state bias, namely, the alternation of bright and dim protrusions in a 3a_0_ (a_0_, silicon surface unit cell of 0.384 nm) periodicity (Fig. 1b), which represent a monomer and a dimer in each trimer unit cell, respectively. As detailed below, this distorted structure is a 1D CDW state as driven by the quasi 1D metallic band of unsaturated dangling bonds of step-edge Si atoms (Fig. 2a).

The silicon trimer chains are well known to contain extrinsic defects, which appear as missing bright protrusions in high bias STM images^39,41^. However, extra local features appear with bright contrast when we lower the bias closer to the Fermi energy where the 3a_0_ periodic modulation in STM becomes weak (Fig. 1a). A careful inspection of this extra feature in the high bias image reveals the presence of a phase mismatch of the 3a_0_ periodicity with units of such as 4a_0_ or 5a_0_ and with gradually decreasing amplitude of the 3a_0_ protrusions (Fig. 1b and Supplementary Fig. 2). These defects are called ×4 or ×5 defects, respectively. Moreover, the hopping of phase defects is frequently noticed by the sudden a_0_ shift of the 3a_0_ modulations (Figs. 1e,f) and its motion is even directly imaged in sequential STM images at 100 K (Fig. 1g and Supplementary Video 1). The enhanced contrast of the phase defects in the low bias suggests the existence of a localized in-gap state. These observations indicate that the trimer Si chains have mobile topological solitons emerging from its 1D CDW states as revealed unambiguously below. Note that the previous observations of the phase defects^39,41,42^ had no means to reveal their intrinsic soliton nature.

### Atomic and electronic structures of mobile phase defects

**Fig. 3:**
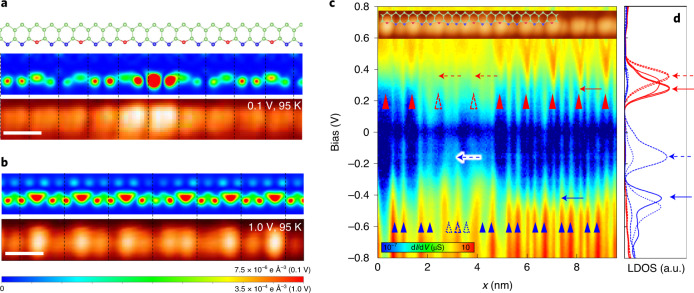
Atomic and electronic structures of a ×4 soliton. **a**,**b** Simulated and experimental STM images at 0.1 V (**a**) and 1.0 V (**b**), respectively (scale bars, 1 nm) for a ×4 phase defect. **c**, STS (d*I*/d*V*) line profile along a Si chain taken at 95 K including a ×4 phase defect. d*I*/d*V* spectra are plotted in a logarithmic colour scale. **d**, The arrows indicate the peak position of the calculated LDOS at the pristine chain (solid lines) and on the defect (dashed lines). Blue (red) lines denote the localized states at undistorted (distorted) Si atoms. The long blue short-dashed line denotes the central (outer) Si atom of the defect that corresponds to the empty (filled) blue triangles in **c**. a.u., arbitrary units.

The undistorted Si step-edge chain has a strongly 1D and partially filled electronic band due to its dangling bond electrons (Supplementary Fig. 8a). In the present structure model, fully relaxed within the DFT calculations (Fig. 1d)^40^, every third Si atom along the step edge is distorted downwards to split the band with an energy gap of 0.6 eV at the Fermi level (Fig. 2a). The band gap is due to the rehybridization of *sp*^3^ dangling bonds into *sp*^2^ and *p* orbitals; the unoccupied *p* bands around 0.2 eV from distorted Si atoms (red balls in Fig. 1d) and the occupied *sp*^2^ bands around −0.4 and −0.7 eV from undistorted Si atoms (blue balls). This electronic structure is consistent with the spectroscopy observation shown in Fig. 3d.

This band structure can be described well with a much simpler 1D tight-binding model considering only the single Si zigzag chain at the step edge (yellow lines in Fig. 2a). The neighbouring Au chains (the bands of dashed lines) affect only the fine structures of the valence bands around −0.4eV to −0.7 eV, which do not affect the following discussion (Supplementary Fig. 11). This 1D tight-binding model is straightforwardly transformed into a trimer Su–Schrieffer–Heeger Hamiltonian as described by three hopping amplitudes (Fig. 2b); *t*_1_ of 0.57 eV, as enhanced by the shorter Si–Si bond length due to the trimer distortion, and the *t*_2_ and *t*_3_ of 0.47 and 0.43 eV, respectively.

Breaking translational symmetry by the trimer structure immediately leads to three degenerated ground states with fractionalized phase shifts of 0, 2π/3 and 4π/3 (Fig. 2b). These ground states can be connected with a few different types of phase defect (or domain walls) with a length of such as 1a_0_, 2a_0_, 4a_0_ and 5a_0_ as shown in Fig. 2c and Supplementary Fig. 7. Only four of them are topologically distinct; a defect with a phase shift of 2π/3 corresponds to the ×2 (2a_0_) or ×5 (5a_0_) defect; a defect with 4π/3 phase shift to a ×1 (1a_0_) or ×4 (4a_0_) defect. To identify detailed atomic and electronic structures of them, we performed DFT calculations with huge supercells (Supplementary Fig. 3). The results reveal that the ×4 structure is most stable in energetics (the formation energy of 0.092 (×4), 0.124 (×5) and 0.177 (×2) eV per unit cell) (Supplementary Table I). The simulated STM image for the ×4 structure reproduces fairly well the experimental ones discussed above, that is, the enhanced contrast at 0.1 V and the shifted protrusions at 1.0 V (Fig. 3a). We also examined the other structure model proposed for the present system, the antiferromagnetic chain model^43^, but the phase defects could not be reproduced consistently (Supplementary Fig. 5).

The DFT and also the tight-binding calculations predict that the ×4 phase defect has its own electronic states within the band gap of the trimer chain as shown in Fig. 3c. The empty and filled states of the pristine 3a_0_ Si chain are located at about +0.3 and −0.5 eV but the phase defect has its localized electronic state at around −0.2 eV. The localized in-gap state is clearly visualized in the STS map on a ×4 phase defect (Fig. 3c). The phase shifts, atomic structures and the in-gap electronic states detailed above converge convincingly to the topological soliton picture of the phase defects observed.

Among four different types of phase defect (Fig. 2c), the ×4 defect occurs most frequently (Supplementary Fig. 1) in accord to the energetics calculated. The ×1 defect is unstable to relax spontaneously into the ×4 defect. The ×2 defect can also easily relax into the ×5 defect by simply recovering one distorted Si atom as shown in Fig. 2c. The energy barrier of this process is 0.01 eV (Supplementary Fig. 15). Even the ×5 defect can transform into a more energetically favourable structure of two ×4 defects combined (called ×4 × 4) as shown in Fig. 2d. The energy barrier is 0.06 eV being smaller than the hopping barrier of about 0.1 eV (Supplementary Fig. 15).

Indeed, we find quite a few ×4×4 defect but rarely a ×5 defect (six ×4×4 and no ×5 defect in total area of 4,140 nm^2^) (Supplementary Fig. 1). Note that the phase shifts themselves are preserved in these relaxation processes of the phase defects. The simulated STM image (Fig. 2f) of a ×4 × 4 defect or a two-soliton bound state is in good agreement with the experiment. Its electronic structure is similar to the isolated ×4 defect in both experiments and calculations (Supplementary Fig. 4) except for a small bonding–antibonding splitting (Supplementary Fig. 3). The merging and splitting of two ×4 defects are hinted in the real time imaging (Fig. 1g and Supplementary Video 1).

### Topological nature and fractional charges

The topological nature of the present system is revealed by analysing its band structure and edge states. The topological invariant of a trimer chain can be related to an effective higher dimensional (2D) bulk system theoretically^15^. We construct such a 2D model by putting an adiabatic dimension and obtain the Chern numbers of (−1, 2, −1) for the three lowest energy bands (Supplementary Fig. 10) as predicted in previous theoretical studies^34,45^. The band gaps of the system contain five different edge states dictated by the topology (Fig. 2e), which match well the DFT calculations (Supplementary Fig. 9). The major edge state of the C phase around 0.2 eV corresponds to the in-gap state observed in the experiment. A 2π/3 or 4π/3 fractional phase shift for an 1D electronic system guarantees fractionalized charges on corresponding solitons, while measuring the charge itself is a tremendous technical challenge; tunnelling spectroscopy uses tunnelling electrons to probe density of states, but does not probe the soliton nature directly. Electronic transport measurements under ultra-high vacuum conditions could provide a more direct probe. In theoretical aspects, we found that the 4π/3 phase-shift soliton has the fractionalized charges of +2e/3 (occupied) and −e/3 (empty) per spin and the 2π/3 phase-shift soliton has +e/3 (occupied) and −2e/3 (empty) per spin (Supplementary Fig. 12 and 13)^35^. The fractional charge is insensitive to detailed domain wall structures but depends only on the phase shift due to its topological origin. For example, the fractional charge on ×5 and ×4 × 4 defects is identical (Supplementary Fig. 13).

### Soliton motions

We observe that the phase defects propagate at a higher temperature. At 90 K, the hopping of solitons (about one hopping for 600 s) is seldom seen, but at 95 K they exhibit about seven hoppings (by one 3a_0_ unit cell of 1.16 nm) within a time window of 600 s (Fig. 1e,f). The hopping becomes more frequent with a small change of the temperature as shown in Fig. 1f (Supplementary Video 1) and solitons become highly mobile already at 115 K. The drift velocity of the soliton at 100 K is measured as 0.10 nm s^−1^, which increases to 0.65 nm s^−1^ at 115 K (Supplementary Fig. 14). An estimation of Arrhenius-type diffusion velocity, *D* = *D*_0_exp(−*E*_b_/*k*_B_*T*), gives the expectation of velocity enhancement of 4.28 from 100 K to 115 K (Supplementary Fig. 15a), which is roughly consistent with the observation. The soliton motion starts at around 100 K, related to the hopping barrier of a soliton 0.1 eV (Supplementary Fig. 15), which is consistent with the thermally induced disordering of the 3a_0_ lattice that was attributed to the generation of phase defects^42^. The real time images also clearly indicate that the soliton is immune to defect scattering (it bounces back or jumps over the extrinsic defects, Fig. 1f) and soliton–soliton scattering (they are reflected but prohibited to pass through: Fig. 1g, Supplementary Video 1 and Supplementary Fig. 16). Of course, when the ground state structure of the Si chain is destroyed, for example, by impurity adsorption and increase of temperature substantially above the onset of its disordering temperature^42^, its edge modes, solitons, cannot be sustained.

### Generation of a single soliton

**Fig. 4:**
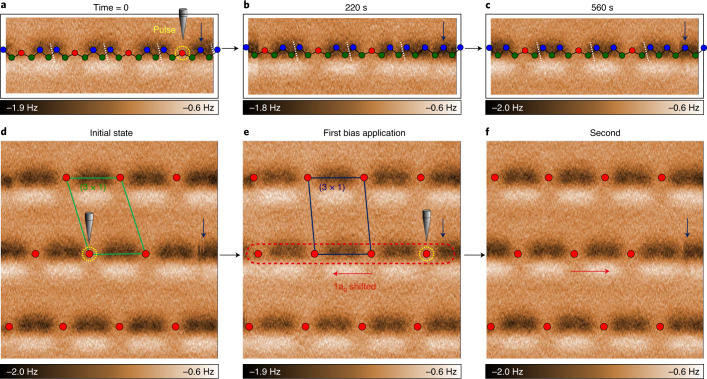
Creation of a single soliton. Frequency-shift non-contact AFM image (4.6 × 1.7 nm^2^) on a Si trimer chain a 4.3 K just before (**a**) and 220 s (**b**) 560 s (**c**) after the injection of the tunnelling pulse (*V* = 0.15 V and *t* = 20 ms) from the metallic probe tip. The excitation and relaxation of the trimer chain are imaged in atomic scale. The atom indicated by the arrow traps the soliton created but is not altered by the tunnelling pulse and is thought to be pinned by a defect or an impurity nearby. **d**–**f** Switching of the selected mid trimer chain structure using on-site bias pulse (4.6 × 4.6 nm^2^). Positions of trimer centre atoms and corresponding single unit cell are indicated for clear comparisons between transitions. Initial state (**d**), first (**e**) and second (**f**) bias application.

We can generate single solitons at low temperature under the probe tip through the application of a voltage pulse. Figure 4a shows an atomically resolved atomic force microscopy (AFM) image of the surface at 4.3 K. In the AFM image, two undistorted Si atoms (blue atoms in the model of Fig. 2) of a trimer appear as a dark contrast due to their closer distance to the tip. After the application of a single tunnelling pulse (0.15 V for 20 ms) at the location of the distorted Si atom (yellow circled in Fig. 4a), one can observe one trimer destroyed (Fig. 4b). This transiently forms a ×6 chain in our structure model (Fig. 2) and relaxes into a ×5 soliton (Fig. 4c) and the phase shift of the neighbouring trimers. This indicates the pair creation of ×1 and ×5 solitons with the former quickly moving out of the view frame to induce the phase shift. The soliton can also be erased by applying the same bias in a nearby site as shown in Figs. 4d–f. That is, the second soliton generated annihilates the first one. This switches the topological phase shift of a given trimer chain back-and-forth, as shown in Figs. 4d–f. That is, one can manipulate a single soliton and decode the topological phase information on each chain (extra data in Supplementary Fig. 17).

### Conclusions

A material realization of a fractionalized soliton has been elusive in an electronic system. Note that the popular dimer solitons have no electronic fractionalization due to spin degeneracy. A close electronic example available is that of phase defects in finite size artificial lattices based on a 2D surface state and adsorbates^46^. However, this system only provides the static modulation of hopping amplitudes for an electronic orbital well away from the Fermi level to preclude the motion and charge fractionalization. That is, these phase defects do not feature the dynamic nature, which is essential to a soliton.

The high mobility of the soliton observed directly here is notable since most of the solitons in previous works on solid surfaces are strongly pinned by defects or strong interchain interaction^30^. Mobile fractional solitons are contrasted with Majorana edge modes, for which an isolated mobile form has not been identified yet. The present solitons are further contrasted with Majorana modes and skyrmions by the fractionalized quanta associated. The soliton–soliton interaction glimpsed here as the formation of a soliton pair has an important implication in quantum information processing to secure an entangled state of solitons^24,25,47–49^. The demonstration of the reproducible creation of an individual soliton here may enable manipulation of such information. Most of the essential ingredients for the exploitation of technological potentials of solitons in electronic systems are secured, such as high mobility, artificial generation/annihilation, switchability^29^ and mutual interaction. One has to overcome the chemical susceptibility of atomic wires and the limited temperate range of their broken symmetry phases for practical applications.

## Methods

### Sample preparations

The Si(553)–Au surface with a regular array of alternating Au chains and Si step-edge chains was fabricated by depositing Au of about 0.5 of a monolayer using a thermal evaporator onto a well cleaned Si(553) substrate at a temperature of 920 K in an ultra-high vacuum. The Si(553) substrate was cleaned by repeated flash heating periods at 1,500 K. The well-ordered array was confirmed by low-energy-electron diffraction and STM images.

### STM measurements

The STM measurements were performed using a commercial low-temperature STM apparatus in an ultra-high vacuum chamber. The system was cooled down by liquid nitrogen while the temperature was carefully controlled by a built-in resistive heater. The measurement was done at various different temperatures between 78 and 125 K at various different biases. The tunnelling current was typically fixed at 30 pA. The STS (d*I*/d*V*) measurement was performed using the standard lock-in technique with a lock-in modulation of 20 mV at 910 Hz and a tunnelling current of 200 pA. For time-dependent topographic measurements for a short segment of a wire (Supplementary Fig. 14a), the time resolution between successive scans was 0.6 s.

### AFM measurements

The imaging of the surface structure manipulation was performed by high-resolution non-contact AFM (nc-AFM) and STM under ultra-high vacuum at 4.3 K using a commercial low-temperature microscope (SPECS GmbH) with simultaneous force-current detection capability. This combined system could effectively decouple the excitation source (tunnelling current) and the local structural probe (AFM) so that the surface structure was investigated precisely by frequency-shift (Δ*f*) images while the tunnelling current was limited to generate transient structures. The Δ*f* images were recorded at a constant height (relative tip elevation defined by the STM imaging set points on the site of step-edge Si chain *V* = +0.5 V and *I* = 160 pA). In the non-contact regime, Δ*f* increased in negative direction as the tip-sample distance got closer, which meant a more protruding structure appeared darker.

### Structure manipulation

Step-edge Si trimer chains can be shifted reproducibly by exciting a local transient structure with the tunnelling current injected. The local transient structure was generated using a bias pulse (+0.15 V for 20 ms) on top of the trimer centre at 4.3 K (the explicit tip operation is described in Supplementary Fig. 18). After sufficient time elapsed, the equilibrium state was achieved by the propagation of the soliton structure along the chain, which led to the phase shift in trimer structure. We found that the initial excitation site, a trimer centre, was prohibited from returning to the same trimer centre, which forced a 1a_0_ lateral shift of the original trimer structure. At a higher temperature than 4.3 K, a higher voltage is expected to induce the locally excited structure or solitons.

### DFT calculations

DFT calculations were performed by using the Vienna ab initio simulation package^50^ within the generalized-gradient approximation using the revised Perdew–Burke–Ernzerhof functional^51^. The Si(553)–Au surface was modelled within periodic supercells with at least four bulklike Si layers and a vacuum spacing of about 12.8 Å. The bottom of the slab was passivated by H atoms. We used a plane-wave basis with a kinetic energy cut-off of 312 eV and a 5 × 2 × 1 *k*-point mesh for the clean Si(553)–Au surface. All atoms except for the bottom two Si layers held fixed at the bulk positions were relaxed until the residual force components were within 0.03 eV Å^−1^. We used large supercells for the defects (roughly 14–17a_0_) and their energetics is summarized in Supplementary Table I. It shows that its relative stability is sound, even though it is difficult to obtain the well-converged isolated energy of solitons due to huge and different-sized supercells. For the tight-binding model calculations, we used the PythTb package by Coh and Vanderbilt^52^.

## Online content

Any methods, additional references, Nature Research reporting summaries, source data, extended data, supplementary information, acknowledgements, peer review information; details of author contributions and competing interests; and statements of data and code availability are available at 10.1038/s41565-021-01042-8.

## Supplementary information

Supplementary Information Supplementary Figs. 1–17 and Table 1.

Supplementary Video 1 Visualized mobile solitons.

## Data Availability

The data that support the findings of this study are available from the corresponding authors on request.

## Extended data

are available for this paper at 10.1038/s41565-021-01042-8.
