# Supplementary material for: Creation and annihilation of mobile fractional solitons in atomic chains
Source: Nat Nanotechnol. 2021 Dec 22;17(3):244–9. doi: 10.1038/s41565-021-01042-8 (PMC8930762; doi:10.1038/s41565-021-01042-8)
Supplement: Supplementary file 1 — Supplementary Figs. 1–17 and Table 1. [file 41565_2021_1042_MOESM1_ESM.pdf]

nature portfolio

https://doi.org/10.1038/s41565-021-01042-8

Supplementary information

Creation and annihilation of mobile fractional solitons in atomic chains

In the format provided by the authors and unedited

Supplementary Information:

Creation and annihilation of mobile fractional solitons in atomic chains

Park et al.

Table of Contents

Supplementary Note 1: Additional information for the STM images

Supplementary Fig. 1. Large area STM images for various defects.

Supplementary Fig. 2. STM line profile of a phase-shifting ×4 defect.

Supplementary Note 2: DFT supercell structures

Supplementary Fig. 3. Supercell structures and local density of states of phase shift defects.

Supplementary Table I Distortion and formation energy of defects.

Supplementary Fig. 4. Comparison between DFT supercell calculations and STM/S data.

Supplementary Note 3: Structural details and tight-binding model

Supplementary Fig. 5. Simulated STM image of the ×4 phase shift defect within the spin-chain model.

Supplementary Fig. 6. Atomic structure and tight-binding parameters.

Supplementary Fig. 7. Schematic images of the 1D zigzag Si chain of the tight-binding model.

Supplementary Fig. 8. 1D electronic band dispersions of the zigzag Si chain at step edge in the DFT and the tight binding model.

Supplementary Fig. 9. Comparison between DFT calculations and the tight-binding model for the phase shift defects.

Supplementary Note 4: Topological properties and fractionality

Supplementary Fig. 10. Topological properties of the open boundary trimer chain.

Supplementary Fig. 11. Effect of the hybridization with a double Au chain attached directly to the Si zigzag chain.

Supplementary Fig. 12. In-gap states and fractionality for long open boundary chains with A-C-B-A and A-C-B-A ground states connected.

Supplementary Fig. 13. In-gap states and fractionality for A-B-A chains.

Supplementary Note 5: Motion of solitons.

Supplementary Fig. 14. Estimation of the soliton velocity.

Supplementary Fig. 15. Propagation and transition energy barriers estimated in DFT calculations.

Supplementary Fig. 16. Reflection and transmission of soliton-soliton scattering.

Supplementary Note 6: Structure manipulation with tunneling current injection

Supplementary Fig. 17. Switching of the selected trimer chain structure using tunneling current.

Supplementary Fig. 18. Tip operation during a single soliton generation.

Supplementary Note 1: Additional information for the STM images

The various defects are found in the large area STM images of the Supplementary Fig. 1 (SFig. 1). The major defect is the ×4 defect (blue ovals) and longer defects (red ovals) are also found as minor species. Most ×4 defects are maintained their own position at 95 K, however, some of them move to different positions (dashed ovals). The ×4 defects seem to be temporarily combined each other to form soliton-soliton pairs (×4×4 and even ×4×4×4, see green ovals). This can be confirmed by the observation that a paired ×4×4 (×4×4×4) defect in SFig. 1a is separated into two ×4 defects (×4 and ×4×4 defect) in a successive scan of SFig. 1b (SFig. 1c). The STM line profile for the major ×4 defect in SFig. 2 clearly indicates that it involves a phase shift and that the amplitude of its protrusions exhibits a typical tangent hyperbolic shape of a soliton around the phase shift.

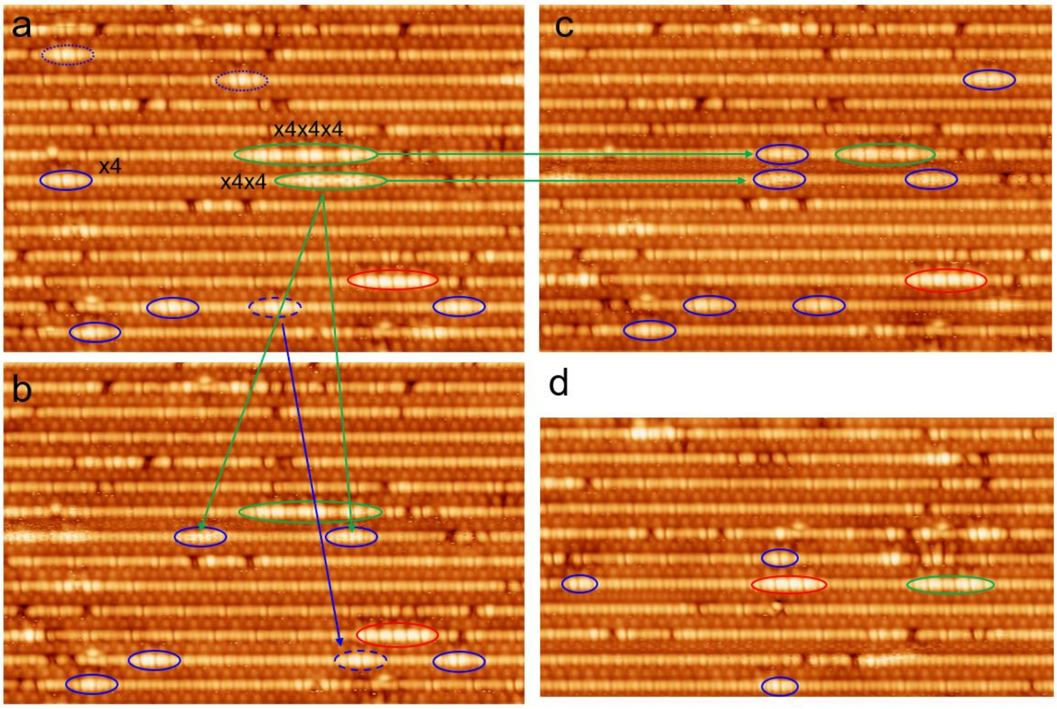

Supplementary Fig. 1. Large area STM images for various defects (V_s = 0.1 V and 95 K) with low energy electronic states manifested by the bright protrusions at low-bias STM images. **a**, **b**, and **c** are the same area scanned successively and **d** is a different area. Blue ovals denote the ×4 defects. Green ovals denote the ×4×4 and ×4×4×4 defects. Red ovals denote other minor type defects. Dashed ovals indicate the mobile ×4 defects.

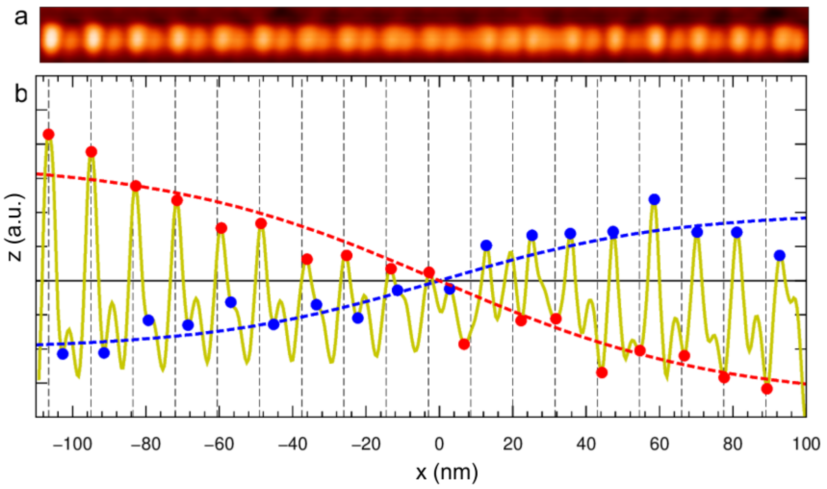

Supplementary Fig. 2. STM line profile of a phase-shifting ×4 defect. **a** STM image (V_s = 1.0 V and 95 K) of a Si step-edge chain with one ×4 defect in its center. **b** Line profile along the center of the Si step edge chain in **a**. Vertical dashed lines denote the $3a_0$ period (11.51 Å). Red and blue circles denote the peak positions of the protrusions in the left and right sides of the phase shift, respectively. Red and blue dashed lines are fitting curves by tangent hyperbolic functions.

Supplementary Note 2: DFT supercell structures

In order to identify the detailed atomic and electronic structure of the phase shift defects, we employed large supercell structures in DFT calculations (SFig. 3). To minimize the soliton-soliton interaction due to the finite supercell size but to make the calculation feasible, we constructed the 9~12a_0 length of pristine domain region. The theoretical LDOS of each supercell in SFig. 3a are shown in SFig. 3b. The in-gap states of the ×2 defect (dashed lines in SFig. 3b) appears at around 0.1 eV as mainly localized on distorted Si atoms of the defect (red balls within the oval in SFig. 3a). The corresponding in-gap states of the ×4, ×5, and ×4×4 defects appear around -0.2 eV originating from the undistorted Si atoms (blue balls within the ovals in SFig. 3a) of the defects. The simulated STM image and their electronic structures are in good agreement with the experimental STM images and STS line profiles as shown in SFig. 4.

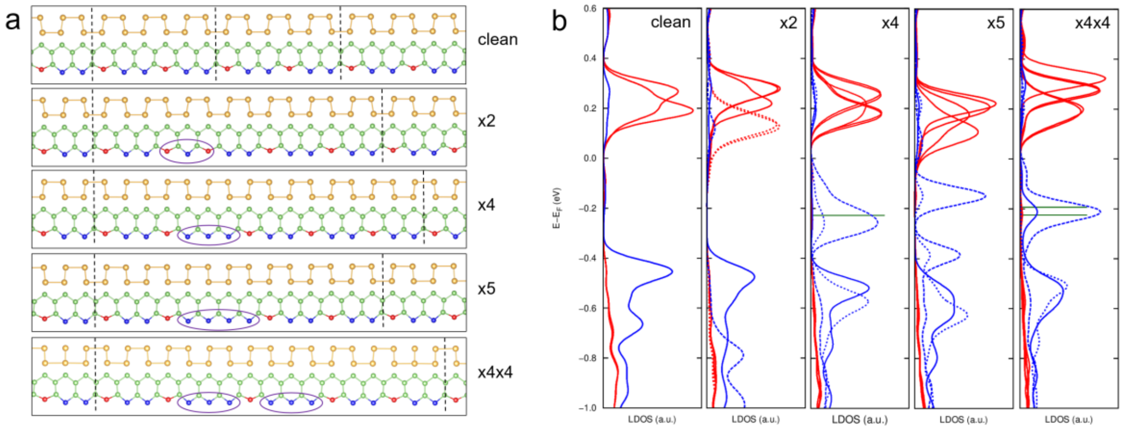

Supplementary Fig. 3. Supercell structures and local density of states of phase shift defects. **a** Atomic structure models in supercell geometries. Dashed lines denote the unit cell and the supercells of 6, 14, 16, 14, and 17a_0 for the pristine surface, ×2, ×4, ×5, and ×4×4 defects, respectively. The defects are marked by ovals. **b** Local density of states calculated. The red and blue solid lines denote the electronic states localized at the blue and red Si atoms of the pristine part of the supercell (the domain region) in **a**. The red and blue dashed lines denote the electronic states on defects. Green lines in the ×4 and ×4×4 defects denote the in-gap states at Γ point indicating the 'bonding-antibonding' energy splitting of the ×4×4 soliton pair. The Fermi level of the ×4 defect is shifted downward by 0.1 eV for better comparison.

Defect	Supercell size	Domain size	$E_{distortion}$ (eV/N)	$E_{formation}$ (eV/unit cell)	Number of solitons (N)
×4	$16a_0$	$12a_0$	0.2456	0.0921	1
×5	$14a_0$	$9a_0$	0.2886	0.1237	1
	$17a_0$	$12a_0$	0.3070	0.1084	1
×2	$14a_0$	$9a_0$	0.4119	0.1765	1
	$17a_0$	$12a_0$	0.4015	0.1417	1
×4×4	$14a_0$	$6a_0$	0.1229	0.1053	2
	$17a_0$	$9a_0$	0.1358	0.0958	2

Supplementary Table I Distortion and formation energy of defects. Distortion ($E_{distortion}$) and formation energy ($E_{formation}$) are defined by $E_{distortion}$ = [$E_{supercell}$ – E_{clean} × (supercell size/unit cell size)]/N and $E_{formation}$ = $E_{supercell}$ × (unit cell size/supercell size) – E_{clean}, respectively, where $E_{supercell}$ and E_{clean} are the total energy of the supercell and clean Si(553)-Au surface, and N is the number of defects in the supercell.

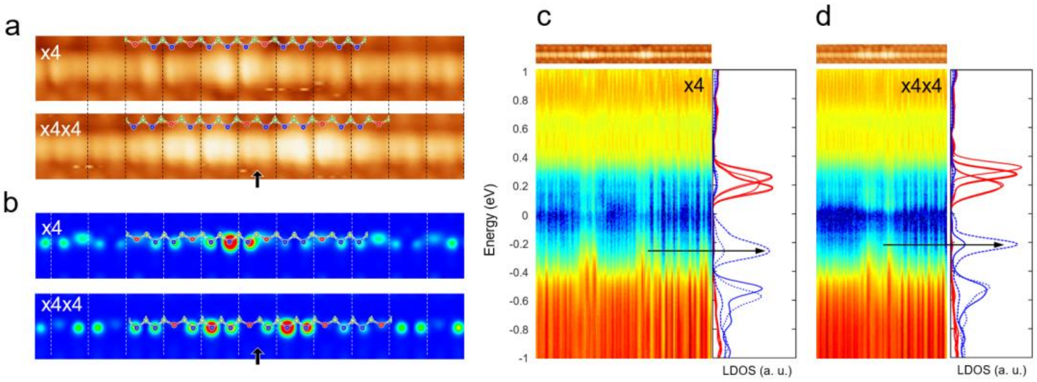

Supplementary Fig. 4. Comparison between DFT supercell calculations and STM/S data. **a** Experimental STM images (V_s = 0.1 V and 95 K) of the ×4 and ×4×4 defects. **b** Simulated STM images for the ×4 and ×4×4 defects as obtained from the partial charge density in DFT calculations (V_s = 0.1 V) at constant height of 2 Å away from the top Si atoms. **c** Line profile STS (dI/dV) data (left) and theoretical LDOS (right) for the ×4 defect. The arrow indicates the peak position of the in-gap states in the theoretical LDOS. **d** Similar comparison for the ×4×4 defect.

Supplementary Note 3: Structural details and tight-binding model

The atomic structure of the Si(553)-Au surface has long been controversial and thus we also examined the solitonic defect structures in the spin-chain model [1], which is another major structural model. This model has a marginal structural distortion in contrast to the model we adopted but a significant spin polarization of the Si dangling bonds (marked red in SFig. 5a) along the step edge with a period of $3a_0$. The simulated STM image at low bias of the ×4 defect based on the spin chain model is comparable to the experimental image (SFig. 5b), however, the enhanced protrusions of the defect center at high bias is distinct from the suppressed protrusions of the experimental image (SFig. 5c).

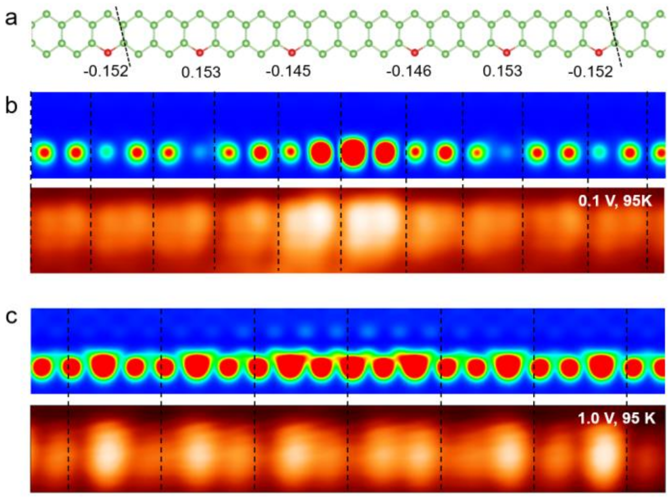

Supplementary Fig. 5. Simulated STM image of the ×4 phase shift defect within the spin-chain model [1]. **a** Atomic structure of the step edge Si chain with a ×4 defect. The numbers denote the local magnetic moment of the spin-polarized Si atoms (red atoms). **b** Simulated STM image (V_s = 0.1 V) as compared with the experimental STM image (V_s = 0.1 V and 95 K). **c** Similar comparison at a high bias of V_s = 1.0 V.

We constructed a tight-binding model for the 1D zigzag Si chain at the step edge based on the recent rehybridized surface structure model (see SFig. 6) [2]. The tight-binding Hamiltonian of the zigzag Si chain with a $3a_0$ periodicity is

$$\begin{aligned} H =& \sum_{n=1}^{N_c}[t_1(c_{1,n}{}^\dagger c_{2,n} + c_{4,n}{}^\dagger c_{5,n}) + t_3(c_{2,n}{}^\dagger c_{3,n} + c_{3,n}{}^\dagger c_{4,n}) + t_2(c_{5,n}{}^\dagger c_{6,n} + c_{6,n}{}^\dagger c_{1,n+1}) + H.c] \\ &+ \sum_{n=1}^{N_c}[U_1(c_{2,n}{}^\dagger c_{2,n} + c_{4,n}{}^\dagger c_{4,n} + c_{6,n}{}^\dagger c_{6,n}) + U_2(c_{1,n}{}^\dagger c_{1,n} + c_{5,n}{}^\dagger c_{5,n}) + U_3 c_{3,n}{}^\dagger c_{3,n}] \end{aligned} \quad (1)$$

Here, $c_i^\dagger$ and c_i are creation and annihilation operators and U_1, U_2, and U_3 are on-site energies.

$$U_1 = 0.68, \quad U_2 = -0.10, \quad U_3 = 0.58\ eV \quad (2)$$

For hopping amplitudes of t_1, t_2, and t_3, we assumed the linear approximation for the change of hopping

amplitudes [3, 4], and the electron-lattice displacement coupling constant α as 4.2 eV/Å to fit the DFT band structure.

$$\begin{aligned} t_1 &= t_0 + \alpha(d_{1,2} - d_0) \\ t_2 &= t_0 + \alpha(d_{2,3} - d_0) \\ t_3 &= t_0 + \alpha(d_{5,6} - d_0) \end{aligned} \tag{3}$$

$$d_{1,2} = d_{4,5} = 2.341, \quad d_{2,3} = d_{4,5} = 2.366, \quad d_{5,6} = d_{6,1} = 2.374 \text{ Å} \tag{4}$$

$$d_0 = 2.339 \text{ Å}, \quad t_0 = -0.58\ eV, \quad \alpha = 4.2\ eV/\text{Å} \tag{5}$$

Finally, we obtained the hopping amplitudes as follow.

$$t_1 = -0.572, \quad t_2 = -0.466, \quad t_3 = -0.433\ eV \tag{6}$$

Three degenerated ground states and six possible phase-shift structures in this tight-binding model are shown in SFig. 7. In the tight-binding model, we assumed strong 1D characters of the zigzag Si chain which can be identified in SFig. 8. The band structure of the pristine structure without the $3a_0$ distortion (SFig. 8a) shows nondispersive bands in the direction perpendicular to the chains (Γ-M) and two parabola-like bands in empty and filled states along the chain direction. The band gap originates from the onsite energy difference between inner and out Si atoms of the zigzag chain. The tight binding bands, including their variation upon the change of the size of the $3a_0$ lattice distortion, are reasonably consistent with the DFT band structure except the hybridized states between the inner Si atoms of the chain and the topmost bulk Si atoms around 1 eV (black circles in SFig. 8a). These states and the inner Si atoms are not crucial in the present discussion. Note that the fully distorted structure of the SFig. 8e is identical to Fig. 2a but it used a reduced number of Si layers (four Si layers) for the bulk. The in-gap states of the phase shift defects (×4, ×2, and ×5) of the open boundary chain are also in good agreement with the DFT results in supercells indicating the validity of the tight-binding model (SFig. 9).

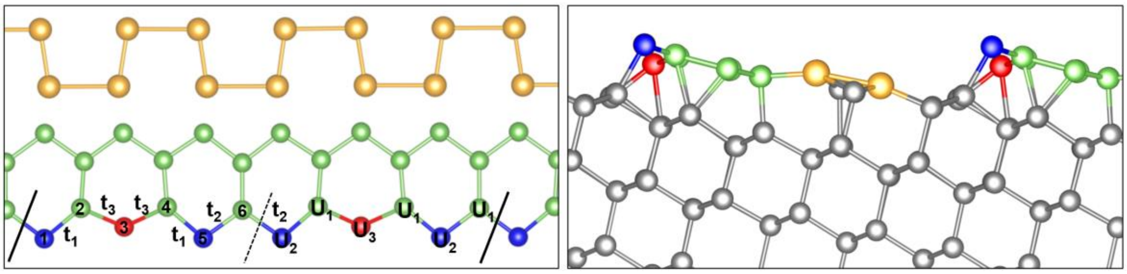

Supplementary Fig. 6. Atomic structure and tight-binding parameters. 1 to 6 are atomic index. t_1, t_2, and t_3 are hopping amplitudes, and U_1, U_2, and U_3 are the onsite energies.

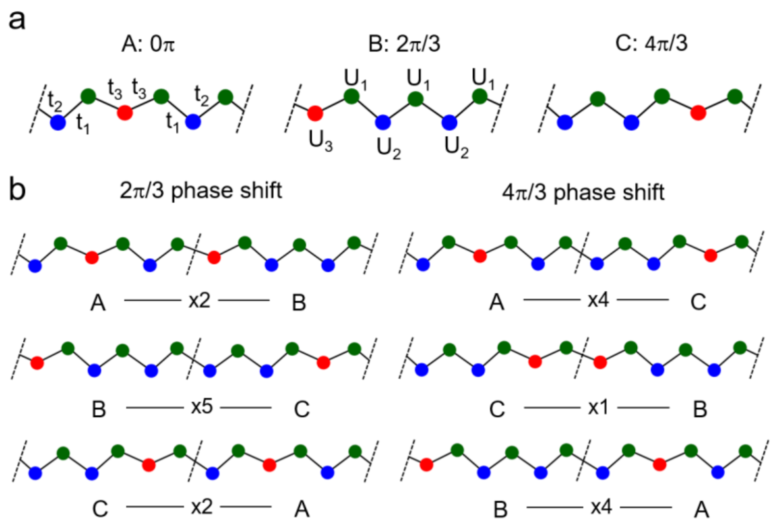

Supplementary Fig. 7. Schematic images of the 1D zigzag Si chain of the tight-binding model. **a** Three degenerated unit cell structures with the $3a_0$ distortion. **b** All six possible phase shift structures. Only four of them are distinct.

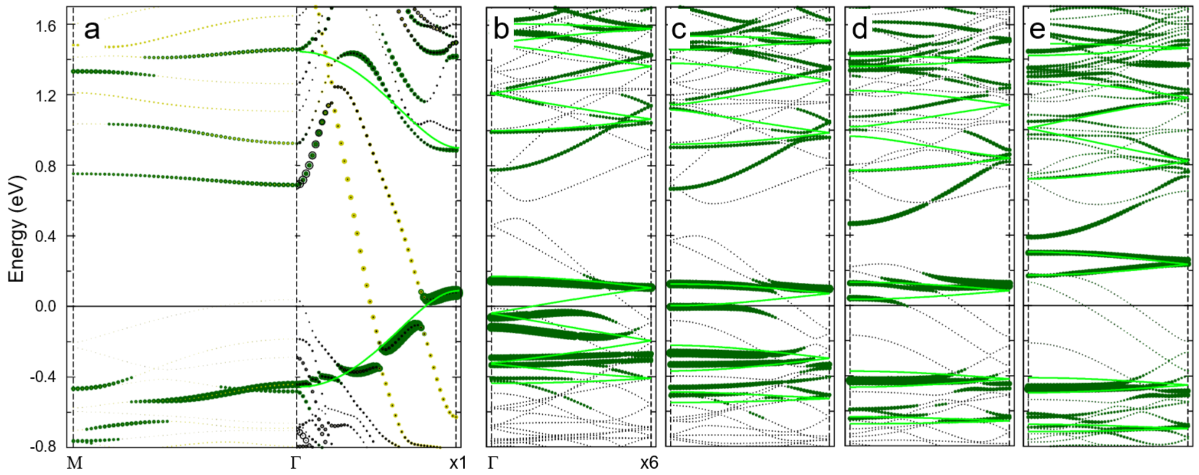

Supplementary Fig. 8. 1D electronic band dispersions of the zigzag Si chain at step edge in the DFT and the tight binding model. **a** Band structures of the undistorted structure. Green (yellow) circles denote the localized states at the zigzag Si chain (the double Au chain) in the DFT calculations. Black circles denote the localized states at the bulk Si atom bonded directly with the inner Si atoms of the zigzag chain. Solid lines denote the tight-binding bands. **b-d** Band structures of the distorted structures as a function of the distortion level (**b** 0.25, **c** 0.5, **d** 0.75, and **e** 1.0). The 0 and 1.0 indicates the undistorted structure and the fully distorted ground state structure. The vertical coordination of the distorted Si atoms is fixed for **b**, **c** and **d**.

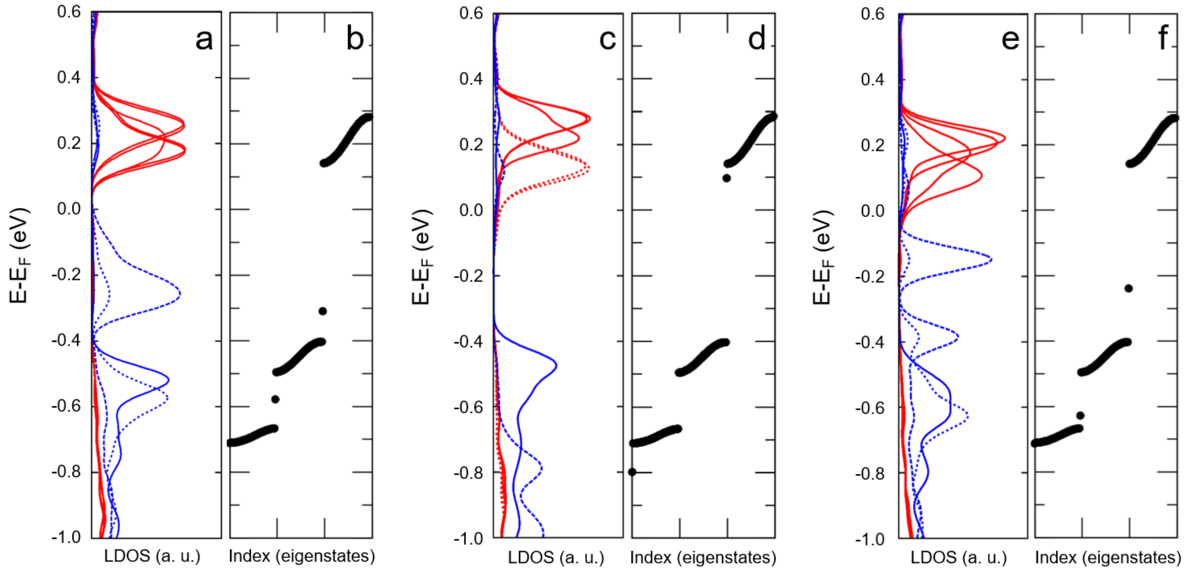

Supplementary Fig. 9. Comparison between DFT calculations and the tight-binding model for the phase shift defects. **a** and **b** for a ×4 defect, **c** and **d** a ×2 defect, and **e** and **f** a ×5 defect. **a**, **c**, and **e** are the local density of states of the DFT supercell calculations and **b**, **d**, and **e** are the tight-binding eigen states of the open boundary chains (50 unit cells). We assumed the onsite energy of 0.2 eV for the undistorted Si atom at the defect region of the ×2 defect to reflect the additional relaxation effect in the DFT calculation.

Supplementary Note 4: Topological properties and fractionality

We provide the detailed analysis of the topological properties of the system in SFig. 10 and further examine the hybridization effect with Au chains in SFig. 11. The open boundary chains for the three degenerated phases display the localized states at both right and left edges within the band gaps depending on their own hopping parameters (SFig. 10a). The right- and left-edge states can also be identified by the adiabatic evolution (red and blue lines in SFig. 10b), which considered a phase evolution along A → B → C → A. We can further validate the topological properties of the simplified current model by constructing a more sophisticated model including the Au chains. As shown in SFig. 11, we constructed a bigger tight binding model, where the zigzag Si chain is directly connected with the double Au chain (without a buffer Si chain, which exist in the real atomic structure). This model has two 1D like bands on the Au chains, which are similar to the full DFT band structure. We considered the interchain hopping strength from 0 to 0.1 in order to reflect the hybridization effect between Au and Si states (SFig. 11c-11e). The optimized value of the Au-Si interchain hopping is 0.05 to best mimic the DFT calculation. Though the edge states of the open boundary chain are marginally hybridized with the Au chain states, the chiral edge states at both ends of the chain appear robustly and their energy levels are not dramatically changed from original ones. These edge states are protected by the inversion symmetry of the trimer chain and have a topological origin [5]. Thus, we can conclude that the topological properties of the phase shift defects are preserved even under the marginal hybridization with Au chains.

In order to identify the fractionality of the phase shift defects, we calculated the quasi-particle number of the tight binding chain [6]. The integrated quasi-particle number N_i is obtained from $N_i = \Sigma_{j<i} (<n_j> - \rho)$, where $<n_j>$ is the electron density of j-th site and ρ is the uniform charge of third filling. Supplementary Fig. 12 shows the in-gap states, their wave functions and the fractional charge of the phase shift defects (the solitons). The solitons carry fractional charges of ±e/3 or ±2e/3 depending only on the phase shift of the defects. That is, different types of defect structures show the same fractional charges (SFig. 13) indicating that the fractionality is insensitive to the detailed atomic structure.

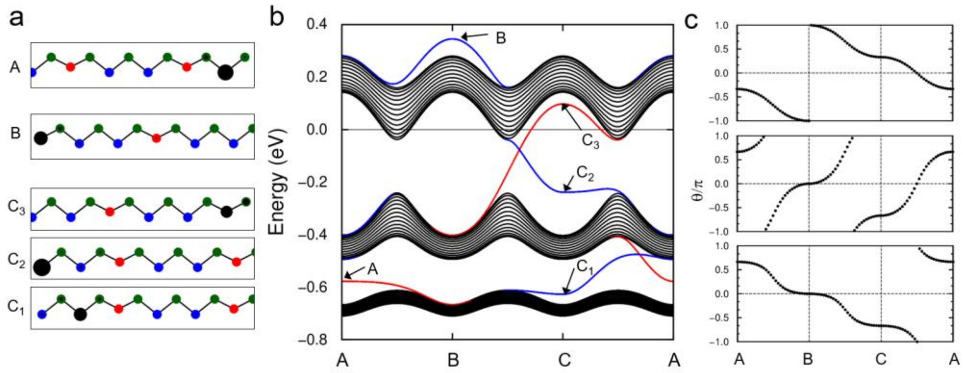

Supplementary Fig. 10. Topological properties of the open boundary trimer chain. **a** Wave functions (black dots) for the edge states with three degenerated phases (A, B, and C) marked by arrows in **b**. **b** Energy spectrum for the adiabatic evolution of three degenerate phases in an energy range between -0.8 eV to 0.4 eV. Tight-binding parameters were taken for the phase evolution of A → B → C → A. The black lines denote the eigen states of the open boundary chain and red and blue lines corresponds to localized edge states at right and left edges, respectively. **c** Evolution of Wannier center (Berry phase) for three individual bands from the bottom to top band.

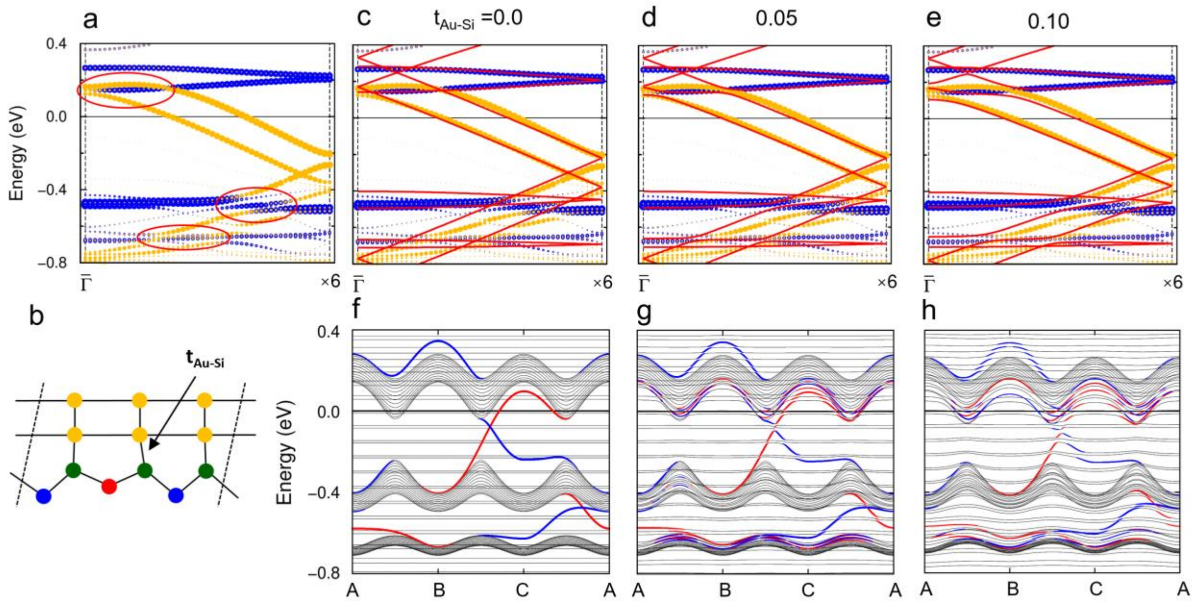

Supplementary Fig. 11. Effect of the hybridization with a double Au chain attached directly to the Si zigzag chain. **a** DFT band structure of the pristine Si(553)-Au surface. The red circles indicate the hybridized states between Au and Si at the band crossing points. Blue and yellow circles denote the localized states at the step edge Si chain and the Au double chain, respectively. The circle size is proportional to the localized states at each chain. **b** Schematics of the tight binding model with a Si zigzag chain and a double Au chain (yellow balls). **c**, **d**, and **e** Tight-binding bands (red lines) as a function of the hybridization strength ($t_{Au\text{-}Si}$). **f**, **g**, and **h** Energy spectrum for the adiabatic evolution of open boundary chains between three different degenerate ground states.

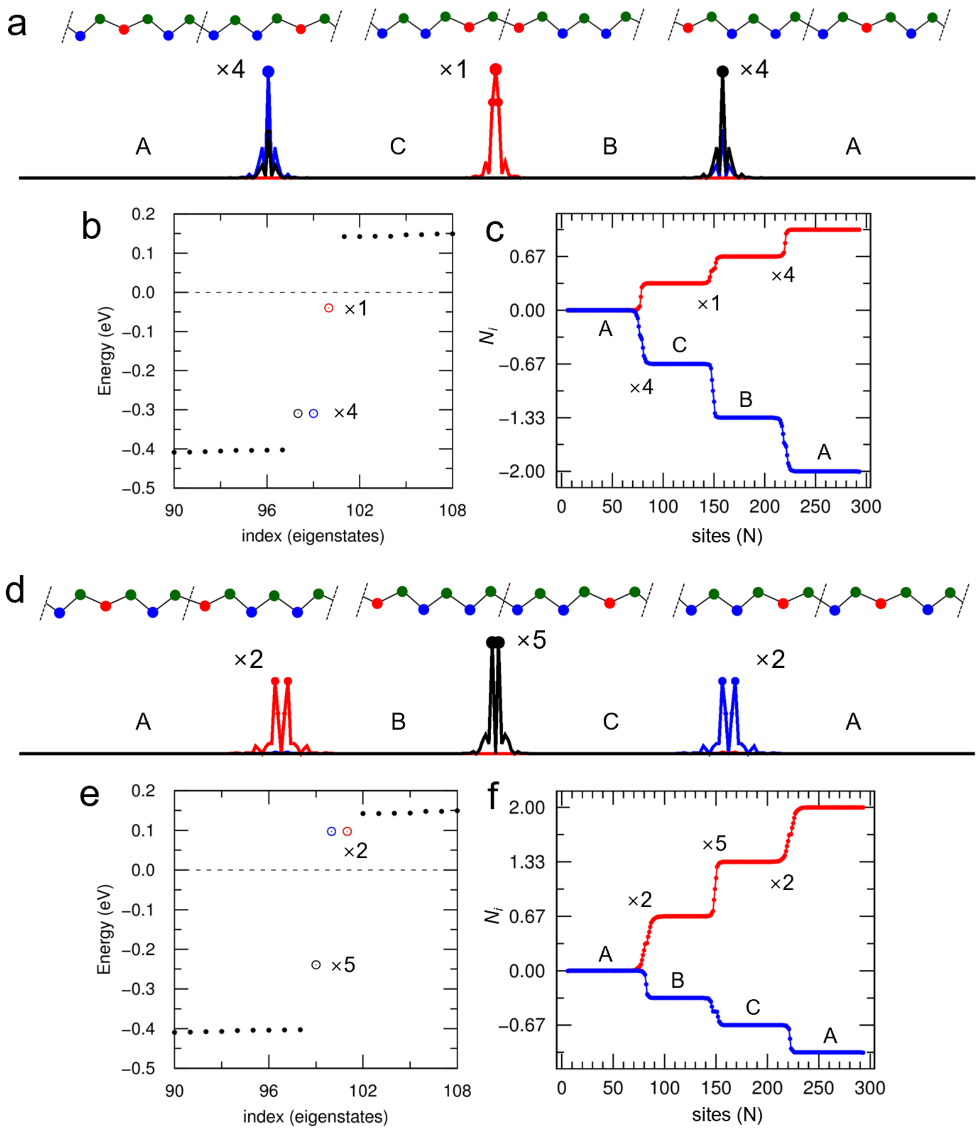

Supplementary Fig. 12. In-gap states and fractionality for long open boundary chains with A-C-B-A and A-C-B-A ground states connected. **a**, **b**, and **c** A-C-B-A chain where each ground state is connected by a 4π/3 defect. **d**, **e**, and **f** A-B-C-A chain connected by 2π/3 defects. **a** and **d** Schematic structures and wave functions of all three defect states. **b** and **e** Energy spectrum with in-gap states. **c** and **f** Integrated quasi-particle number calculated. Red and blue lines represent the unit cell averaged quasi-particle number for the empty (+e for **c** and +2e for **f**) and occupied states (-2e for **c** and -e for **f**).

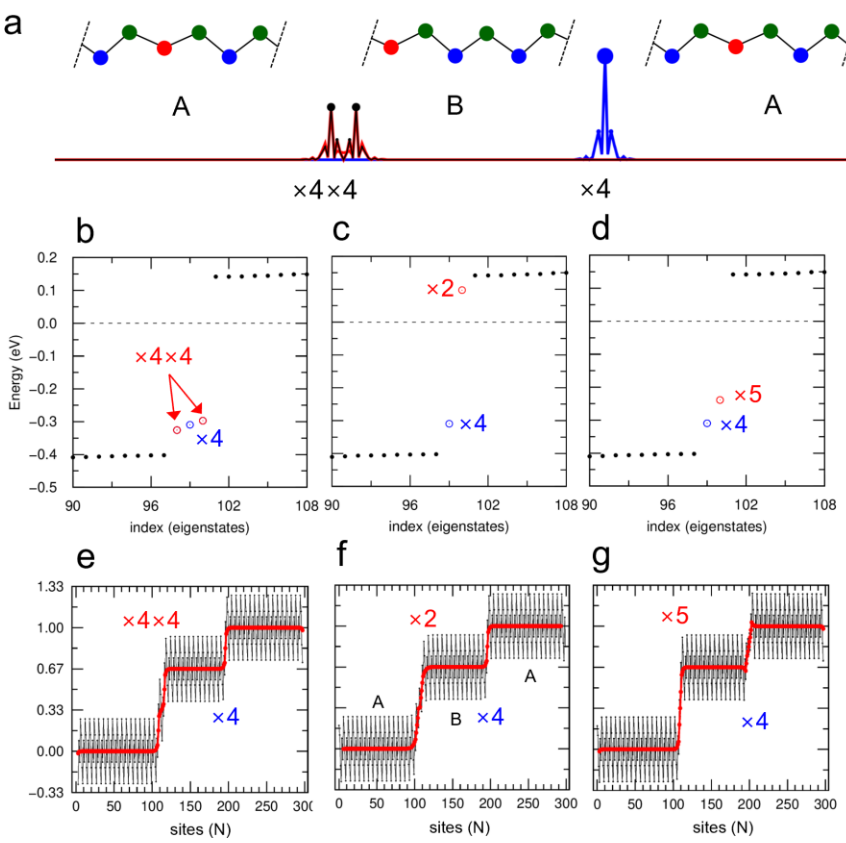

Supplementary Fig. 13. In-gap states and fractionality for A-B-A chains where A and B ground states are connected by three different types of defects with a 2π/3 phase shift and one ×4 defect. **a** Schematic structures and wave functions of the open boundary chain with ×4 and ×4×4 defect states. **b-d** Energy spectrum of three different 2π/3 defects (**b** ×4×4, **c** ×2, and **d** ×5). **e-g** Integrated quasi-particle number for each chain (**e** ×4×4, **f** ×2, and **g** ×5). Red line represents the unit cell averaged quasi-particle number for the empty states (+e).

Supplementary Note 5: Motion of solitons.

Supplement Figures 14 and 15 show the estimation of the soliton velocity and the theoretical energy barrier for the soliton hopping, respectively. The propagation energy path for the ×4 defect is shown in the inset of SFig. 15a: One undistorted Si atom at the defect (one distorted Si atom at domain boundary) is distorted (recovers) as marked by dashed box and, consequently, the ×4 defect is propagated by $3a_0$ with an energy barrier of about 0.1 eV. The propagation energy barrier of the ×5 defect is about 0.1 eV, but it prefers a transition into two ×4 defect in energetics (SFig. 15b).

Supplement Figure 16 shows a schematic image of the soliton-soliton scattering process. The ×4 solitons have to move in $3a_0$ steps on the ordered chain with a $3a_0$ periodicity and, in this circumstance, the soliton-soliton transmission process is prohibited by the $3a_0$ order of the host chain. To preserve the ×4 soliton in the transmission process, the phase of whole chain has to be shifted or an additional shift (by $\pm a_0$, see open green and yellow circles in SFig. 16) of the ×4 soliton is required. The latter is simply identical to the reflection process. Thus, the reflection and the transmission can be distinguished by the change of the boundary condition.

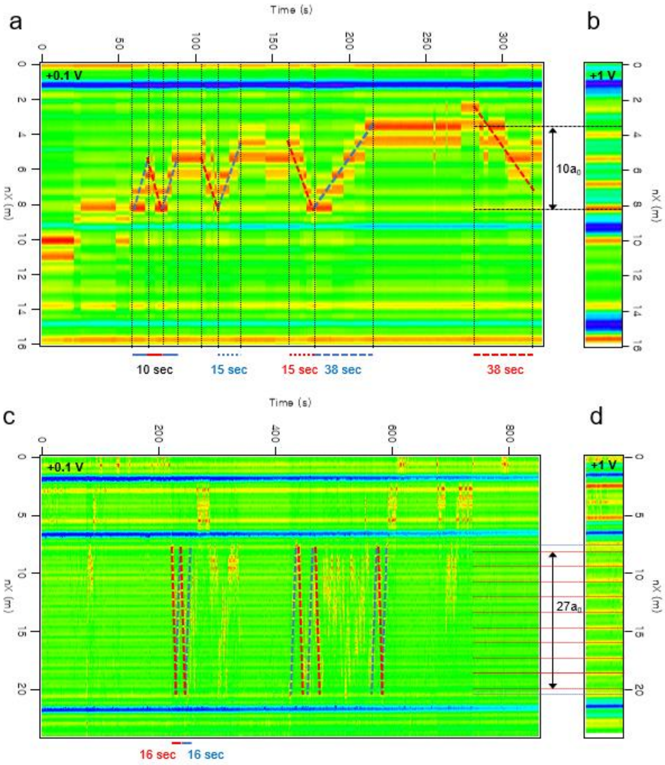

Supplementary Fig. 14. Estimation of the soliton velocity. Soliton motion as captured in the series of STM line scans (V_s = 0.1 V) along a chain segment (the vertical axis). The blue contrasts indicate the impurity sites confining the solitons and the red contrasts represent the soliton position. A representative line scan is shown at the vertical bar in the right panel. **a** and **b** A soliton travels a distance of $10a_0$ or 3.84 nm (V_s = 1 V) at 100 K. **c** and **d** A soliton travel a distance of $27a_0$ or 10.37 nm at 115 K and is scattered back and force by the two confining impurities. From time to time, the soliton jumps into the neighboring segment in the upper part. The estimated velocities are 0.232 and 0.648 nm/sec at 100 and 115 K, respectively.

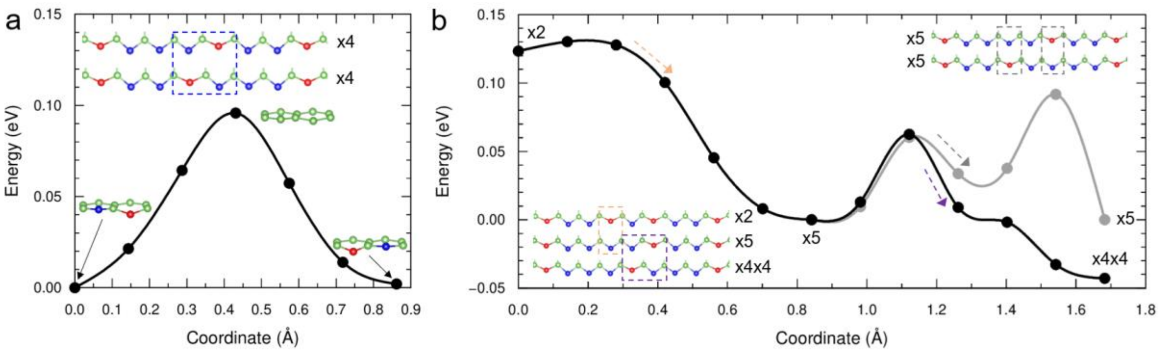

Supplementary Fig. 15. Propagation and transition energy barriers estimated in DFT calculations. **a** Propagation energy barrier for the ×4 defect. **b** Transition energy barrier from the ×2 to ×4×4 through a ×5 defect (black line). That is, these soliton changes its structure during propagation. Gray line denotes the propagation energy barrier for the ×5 defect.

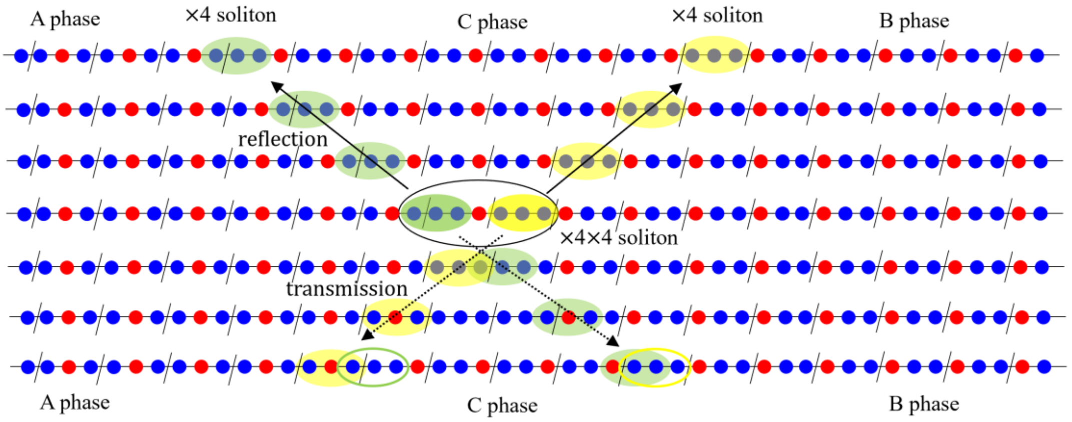

Supplementary Fig. 16. Reflection and transmission of soliton-soliton scattering. Red and blue balls represent the distorted and undistorted Si atoms of the zigzag Si chain. Solid lines are the $3a_0$ unit cell. The left and right ×4 defects are marked by green and yellow shades, respectively. Solid and dashed arrows indicate the reflection and transmission process after the soliton-soliton scattering. The open green and yellow circles indicate required additional shift to preserve the ×4 solitons in the transmission process. This indicates that the transmission is prohibited by the boundary condition.

Supplementary Note 6: Structure manipulation with tunneling current injection

Supplementary Figure 17 shows additional examples of step-edge structure manipulation at 4.3 K (#1 from Fig. 4 in the original text is provided here for comparisons). For all frequency shift (Δf) images at constant height, tip-sample distance corresponds to the relative elevation defined by the STM imaging set points on the site of step-edge Si chain V=+0.5 V and I=160 pA. At this experimental condition, tunneling bias higher than +0.15 V was observed to relieve the $3a_0$ periodic distortions of the Si trimer chains. Single bias pulse condition (V=+0.15 V and t=20 ms) for the manipulation was determined based on this minimum bias requirement for the excited structure (SFig. 18).

For the different experimental condition (temperature, tip height, etc.), optimized bias pulse is expected to be determined by finding out the minimum bias requirement at that condition.

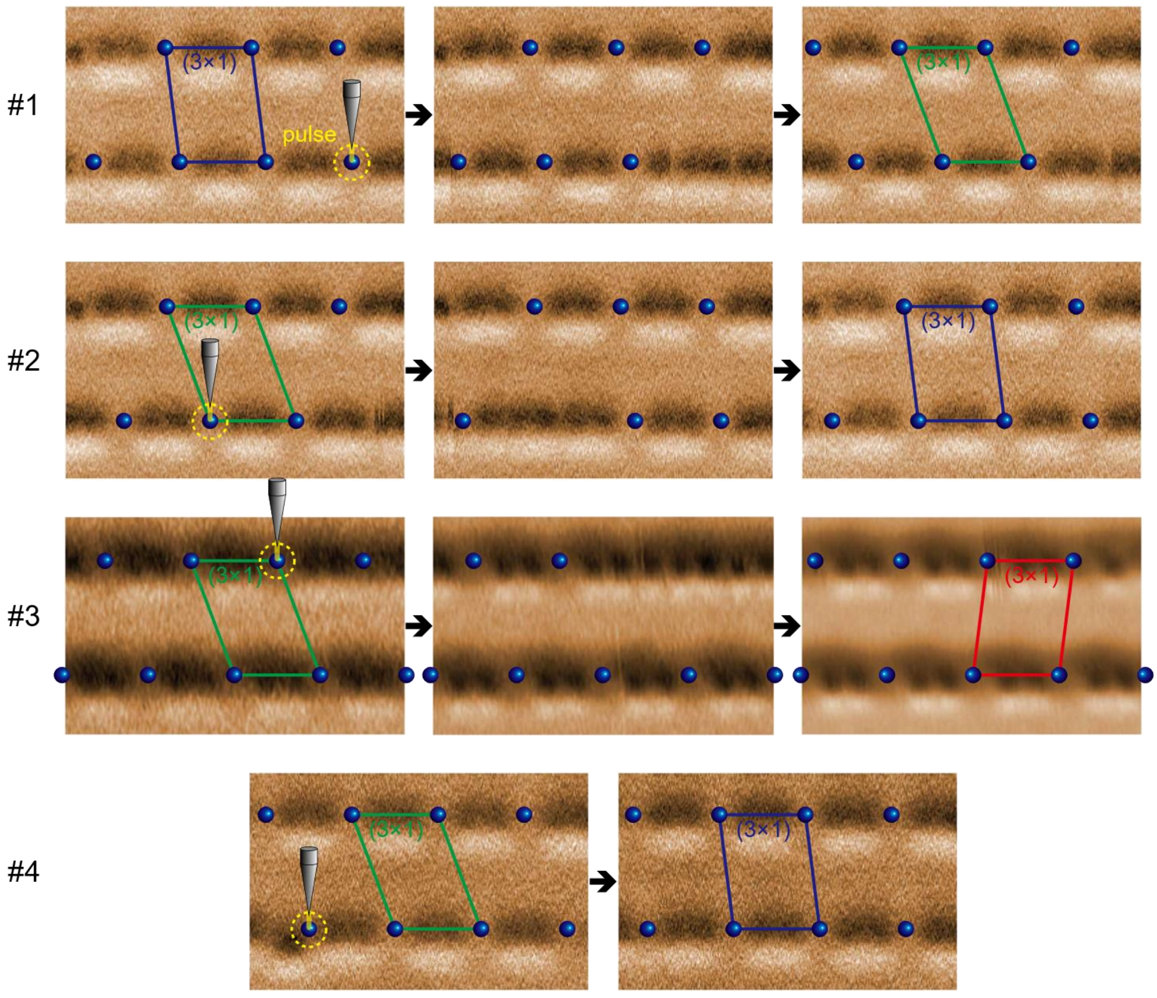

Supplementary Fig. 17. Switching of the selected trimer chain structure using tunneling current. Frequency shift noncontact atomic force microscopy images (4.6×2.9 nm^2) on selected Si trimer chains at 4.3 K before and after the injection of the tunneling pulse (V=0.15 V and t=20 ms) from the metallic probe tip. Positions of trimer center atoms and corresponding single unit cells are indicated for clear comparisons between transitions.

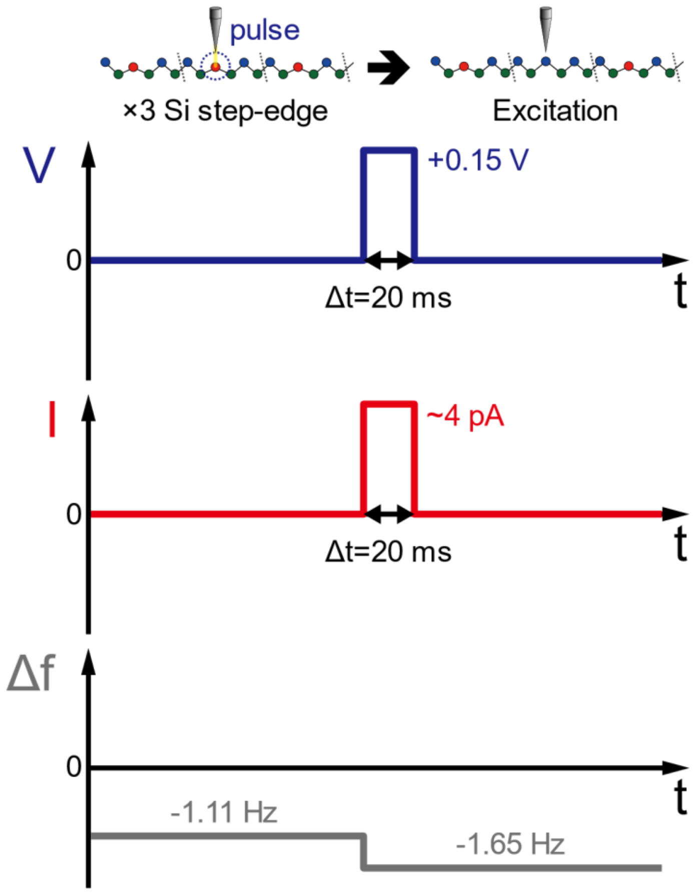

Supplementary Fig. 18. Tip operation during a single soliton generation. Tip was located on the one of trimer center atoms at constant height. Applied bias was zero except for the moment of current injection (V=+0.15 V and I~4 pA) for 20 ms. The bias pulse relieved the ×3 step-edge structure, which pulls the down-distorted center atom upwardly (change in Δf reflects this fact).

Supplementary References

[1] S. C. Erwin and F. J. Himpsel, Nat. Commun. **1**, 58 (2010).

[2] C. Braun, U. Gerstmann, and W. G. Schmidt, Phys. Rev. B **98**, 121402(R) (2018).

[3] W. P. Su, J. R. Schrieffer, and A. J. Heeger, Phys. Rev. Lett. **42**, 1698 (1979).

[4] W. P. Su, J. R. Schrieffer, and A. J. Heeger, Phys. Rev. B **22**, 2099 (1980).

[5] V. M. Martinez Alvarez, M. D. Coutinho-Filho, Phys. Rev. A **99**, 013833 (2019).

[6] D. González-Cuadra, A. Dauphin, P. R. Grzybowski, M. Lewenstein, and A. Bermudez, Phys. Rev. B **102**, 245137 (2020).
